# Supplementary material for: Cannabidiol improves learning and memory deficits and alleviates anxiety in 12-month-old SAMP8 mice
Source: PLoS One. 2025 Aug 14;20(8):e0296586. doi: 10.1371/journal.pone.0296586 (PMC12352845; doi:10.1371/journal.pone.0296586)
Supplement: S1 File — (DOCX) [file pone.0296586.s001.docx]

**Supplement**

**Table 1.**

**CBD**

**Potency Test Results – WI-001 HPLC -UV**

**TEST Concentration (mg/g)**

CBD 817.00

CBG <1.00

CBD-A <1.00

CBN <1.00

Delta 9 THC 35.30

Delta 8 <0.10

CBC 29.70

THC-A <1.00

THC-V <1.00

**Total 882.00 (mg/g)**

**Terpene Test Results – GCMS**

**TEST Microgram Per Gram (ug/g) TEST Microgram Per Gram (ug/g)**

Alpha-Pinene 7908.13 Linalool 2251.64

Camphene 510.21 Fenchyl Alcohol 862.94

Sabinene N.D. Borneal 231.70

Beta-Pinene 600.30 Alpha Teripineol 1584.19

Beta-Myrcene 36124.97 Gamma Teripineol N.D.

Alpha-Phellandrene 364.37 Beta-Caryophyllene 14293.41

Delta-3-Carene N.D. Elemene 426.54

Alpha-Terpinene 218.14 Alpha-Humulene 4249.53

P-Cymene 350.17 Valencene N.D.

Limonene 3740.29 Cis-Nerolidol N.D.

Eucalyptol N.D. Trans Nerolidol 1346.66

Ocimene 1116.97 Caryophyllene Oxide 271.23

Gamma-Terpinene 136.07 Guaiol N.D.

Sabinene Hydrate N.D. Alpha-Bisabolol 12594.15

Terpinolene 3264.22

Fenchone 180.08

**Total Terpenes 92625.91 (ug/g)**

**Table. 2. qPCR primer sequences.**

| **Gene** | **Forward Primer** | **Reverse Primer** |
| --- | --- | --- |
| 36B4 | CACTGGTCTAGGACCCGAGAAG | GGTGCCTCTGAAGATTTTCG |
| HPRT | gcctaagatgagcgcaagttg | tactaggcagatggccacagg |
| PPIB | GGCTCCGTCGTCTTCCTTTT | ACTCGTCCTACAGATTCATCTCC |
| SOD1 | gcccggcggatgaaga | cgtcctttccagcagtcaca |
| SOD2 | Ctctggccaagggagatgtt | gtcccccaccattgaacttc |
| CAT | ccgagtctctccatcaggttt | tcatgtgccggtgaccat |
| GPX3 | aactcggagatactccccagtct | gctggaaattaggcacaaagc |
| SCD1 | ccggagaccccttagatcga | tagcctgtaaaagatttctgcaaacc |
| TXNRD1 | CCCACTTGCCCCAACTGTT | GGGAGTGTCTTGGAGGGAC |
| TXN1 | tgtggtgttccttgaagtggat | ggcatgcatttgacttcacagt |
| GSTM2 | ACACCCGCATACAGTTGGC | TGCTTGCCCAGAAACTCAGAG |
| PRDX3 | tggattcccacttcagtcatct | tggcccaaaccaccattc |

**Figure S.1**


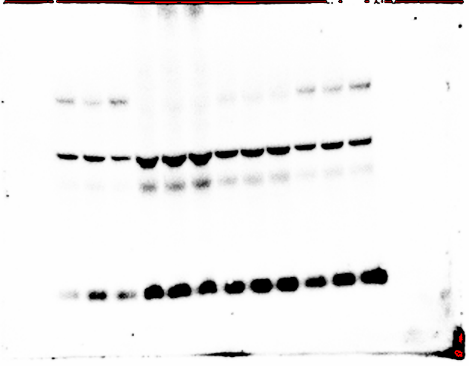

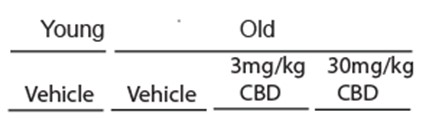

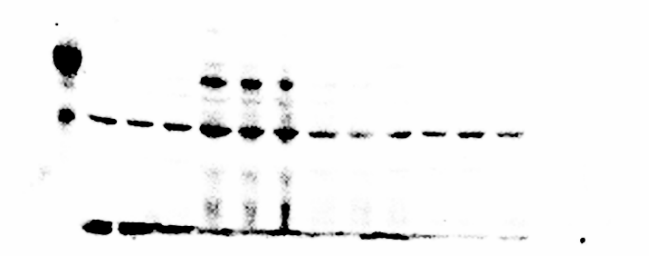

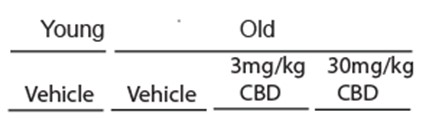

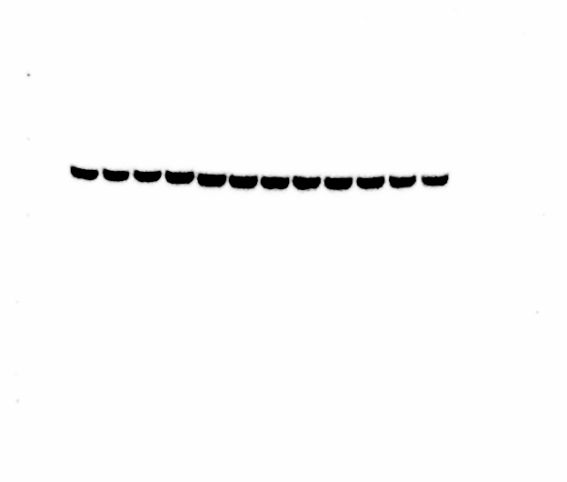

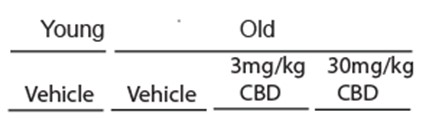


90KD

HSP90

gp91^phox^

4HNE

65KD

67KD

60KD

140KD

40KD

75KD

52KD

80KD
